# Mesoscale morphology at nanoscale resolution: serial block-face scanning electron microscopy reveals fine 3D detail of a novel silk spinneret system in a tube-building tanaid crustacean

**DOI:** 10.1186/s12983-016-0146-0

**Published:** 2016-03-22

**Authors:** Tomonari Kaji, Keiichi Kakui, Naoyuki Miyazaki, Kazuyoshi Murata, A. Richard Palmer

**Affiliations:** Systematics and Evolution Group, Department of Biological Sciences, University of Alberta, Edmonton, AB T6G 2E9 Canada; Allgemeine & Spezielle Zoologie, Institut fuer Biowissenschaften, Universität Rostock, Rostock, 18055 Germany; Department of Biological Sciences, Faculty of Science, Hokkaido University, Sapporo, 060-0810 Japan; National Institute for Physiological Sciences, Okazaki, Aichi 444-8585 Japan

**Keywords:** Renaissance of morphology, High-resolution imaging, Three-dimensional imaging, Serial block-face scanning electron microscope, Silk gland morphology, Thread secretion, Comparative morphology, Tanaidacea, Arthropoda

## Abstract

**Background:**

The study of morphology is experiencing a renaissance due to rapid improvements in technologies for 3D visualization of complex internal and external structures. But 3D visualization of the internal structure of mesoscale objects — those in the 10–1000 μm range — remains problematic. They are too small for microCT, many lack suitable specific fluorescent markers for confocal microscopy, or they require labor-intensive stacking and smoothing of individual TEM images. Here we illustrate the first comprehensive morphological description of a complete mesoscale biological system at nanoscopic resolution using ultra-modern technology for 3D visualization — serial block-face scanning electron microscopy (SBF-SEM). The SBF-SEM machine combines an in-chamber ultramicrotome, which creates a serial array of exposed surfaces, with an SEM that images each surface as it is exposed. The serial images are then stacked automatically by 3D reconstruction software. We used SBF-SEM to study the spinneret (thread-producing) system of a small, tube-dwelling crustacean that weaves tubes of silk. Thread-producing ability is critical for the survival of many small-bodied animals but the basic morphology of these systems remains mysterious due to the limits of traditional microscopy.

**Results:**

SBF-SEM allowed us to describe — in full 3D — well-resolved components (glands, ducts, pores, and associated nerves and muscles) of the spinneret system in the thoracic legs and body segments of *Sinelobus* sp. (Crustacea, Peracarida, Tanaidacea), a tube-building tanaid only 2 mm in body length. The 3D reconstruction by SBF-SEM revealed at nanoscale resolution a unique structure to the gland and duct systems: In each of three thread-producing thoracic segments, two separate ducts, derived from two separate glands located in the body, run through the entire leg and merge at the leg tip just before the spinneret pore opening. We also resolved nerves connecting to individual setae, spines and pores on the walking legs, and individual muscles within each leg segment.

**Conclusions:**

Our results significantly expand our understanding of the diversity of spinneret systems in the Crustacea by providing the first well-resolved view of spinneret components in the peracarid crustacean order, Tanaidacea. More significantly, our results reveal the great power of SBF-SEM technology for comprehensive studies of the morphology of microscopic animals.

**Electronic supplementary material:**

The online version of this article (doi:10.1186/s12983-016-0146-0) contains supplementary material, which is available to authorized users.

## Background

Morphology has been considered a distinct scientific discipline since it was first named by the 18th century German poet, Johann Wolfgang von Goethe [1]. Although it contributed significantly to our understanding diversity and evolution [2], comparative morphology was largely overshadowed by the rise of experimental embryology in the 19th century [3]. However, in recent decades major progress has been made on visualization techniques that permit complex forms to be described in full 3D. Both confocal laser scanning microscopy (cLSM) and micro-computer tomography (micro-CT), have facilitated a “renaissance of morphology” [4, 5] as a crucial discipline in the biological sciences. However, even with these new technologies, three-dimensional data are still difficult to acquire for non-specific (unlabelled) meso-scale objects (roughly 10–1000 μm) in a sufficient resolution (see [6]), even though this size range is essential to understanding the diversity and evolution of many animal groups that include small-bodied members [7].

Several approaches to 3D reconstruction using electron microscope images have been developed in recent decades [8]. For example, “serial-section scanning electron microscopy” [9], where SEM images of serially collected ribbons of ultra-thin sections were aligned and stacked manually. Although this method enabled visualization of large-volume specimens at nano-scale resolution, it was generally very time consuming. The “focused ion beam scanning electron microscopy” is a fully automated image acquisition system using a focused ion or plasma beam to mill away ultrathin sections of resin-embedded specimens in the SEM chamber. It is one of the quickest methods for EM-level 3D reconstruction, but it is commonly only used to visualize cellular level structures due to the limited field of view [8]. Serial block-face scanning electron microscopy (SBF-SEM), the method used here, uses a robotic ultramicrotome-embedded within a scanning electron-microscope. It offers a significant advance in fully automated three-dimensional reconstruction of meso-scale structures to be observed at nanometer resolution [8, 10]. The machine was developed mostly for medical and cell-biology applications to re-construct three dimensional nanostructure of cellular and neuronal structures [11]. However, few biologists seem to realize that this technology offers great power for studying whole-body morphology of meso-scale organisms or objects [6, 12].

Here we showcase how SBF-SEM technology can reveal the detailed internal anatomy of muscles, nerves, glands, and ducts — in full 3D — and show their relation to external features (limb segments, joints, setae and pores) in animals otherwise difficult to study because of their small body size. These observations allow us to answer previously intractable questions: a) What is the complete morphology of the spinneret (thread-producing) system and associated structures in the legs of a tiny benthic crustacean? and b) How does its form compare to thread-producing systems in other Crustacea?

Thread-producing species have evolved independently in many different crustacean groups, and they exhibit a variety of spinneret systems [13]. The Tanaidacea, where adults typically bear a pair of remarkable claws and show a well-developed tube-dwelling behavior, is one of the most thread-dependent taxa in the Crustacea. Threads are widely used to anchor the body to the substratum and to construct flexible tubes within which they dwell. The spinneret system has therefore likely played a key role in tanaid diversification [13]. Unfortunately, the knowledge of tanaidacean spinneret system remains limited because, except for a few partial descriptions by traditional microscopy and histological sectioning — [14] and [15] for Paratanaoidea, [13] for Kalliapseudidae, — no descriptions exist for any species of Tanaoidea, a diverse and well-known thread-dependent superfamily. A complete morphological description has proved elusive because of the complex and intricate arrangement of the glands and nanoscopic ducts, and because of the inability to label these structures with fluorescent dyes, which would permit cLSM visualization.

We therefore provide the first comprehensive morphological description of all the elements in the spinneret system of the tanaid *Sinelobus* sp (Tanaoidea) at ultra-high resolution using SBF-SEM. The pereopods (walking legs) in this species show a clear functional division between the anterior half and posterior half of the body. Pereopods 1–3 possess complete spinneret function, which is lacking in pereopods 4–6. These functional differences also raise an interesting question about how the morphology of spinneret-bearing legs differs from non-spinneret-bearing legs.

## Methods

An undescribed species of *Sinelobus* sp. was collected from Hagi (Yamaguchi Prefecture, 34°26'10.1"N 131°25'08.4"E), then maintained in breeding colonies in the laboratory for more than 1 year. Several intact specimens of *Sinelobus* sp. fixed in 70 % ethanol were deposited as voucher specimens in the Hokkaido University Museum, Sapporo, Japan (catalogue number ICHUM-5114).

To prepare specimens for SBF-SEM, individuals were fixed with 2 % glutaraldehyde and 2 % paraformaldehyde in 0.15 M cacodylate sodium buffer with 2 mM CaCl_2_ (pH 7.4) for 5 h at 4 °C, then decalcified in 10 % EDTA in water for 2 days at 4 °C. The specimens were post-fixed with 2 % osmium tetroxide and 1.5 % potassium ferrocyanide in the same buffer for 2 h at room temperature. They were incubated in 1 % thiocarbohydrazide for 30 min at room temperature, and fixed again with 2 % osmium tetroxide in water for 1 h at room temperature. *En bloc* staining was performed with 1 % uranyl acetate for 3 h at room temperature and then with Walton’s lead-aspartate solution (20 mM, pH5.5) for 60 min at 60 °C. The specimens were washed with cacodylate buffer or distilled water between each step described above. Each specimen was a) dehydrated by a graded ethanol series (30–100 %) at 4 °C with 30 min for each step, b) transferred to 100 % acetone for 1 h, and c) incubated in a graded Durcupan resin series (25, 50, 75, 100 % using acetone as a solvent) in a vacuum chamber for 12 h at each step. The resin was allowed to polymerize at 60 °C for 3 days. Trimmed resin blocks were glued onto an aluminum SBF-SEM rivet with conductive epoxy resin (SPI Conductive Silver Epoxy; SPI Supplies and Structure Prove, Inc., West Chester, PA, USA), and coated with gold using an ion coater. Scanning electron microscopes (SIGMA/VP and MERLIN, Carl Zeiss Microscopy, Jena, Germany), equipped with an in-chamber ultramicrotome system and a back-scattered electron detector (3View; Gatan Inc., Pleasanton, CA, USA), were used to slice and image each specimen as described previously [16]. The serial-section image stack was acquired in an automated fashion by using Gatan Digital Micrograph software. The image stack was automatically aligned using the registration plug-in “Register Virtual Stack Slices” in Fiji/ImageJ software package ([17] available from http://fiji.sc/Fiji). Three-dimensional reconstructions were performed using IMARIS 6.4.0 (Bitplane AG, Zurich, Switzerland). For normal SEM observation (SIGMA/VP, Carl Zeiss Microscopy, Jena, Germany), specimens were fixed using 2 % glutaraldehyde; dehydrated using a standard ethanol dehydration series; freeze-dried; and coated with gold using an ion coater.

## Results and discussion

**Fig. 1:**
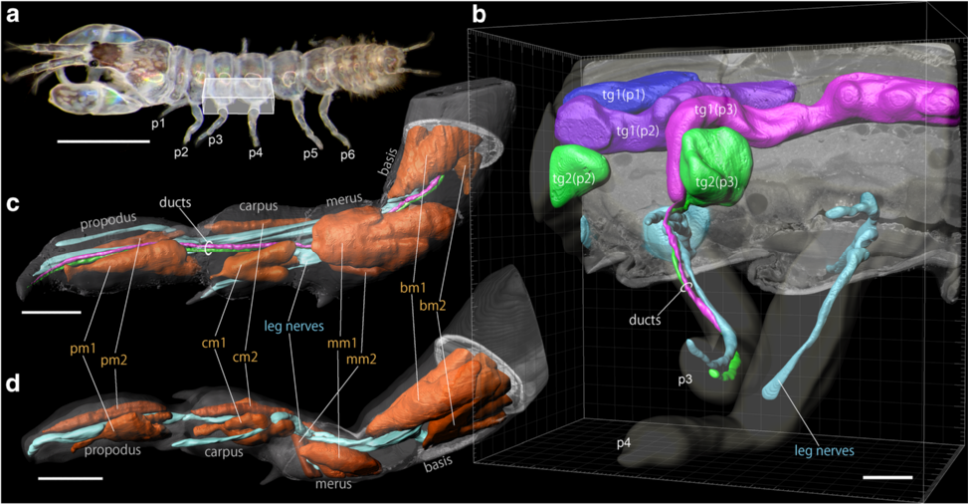
Overall morphology of glands, ducts, muscles and nerves in the legs and associated body regions of male *Sinelobus* sp., as revealed by SBF-SEM scans. **a** Whole animal; the box indicates the area scanned in **b. b** left-half of pereonites 3–4 with legs attached (see Additional file 1 for 3D movie). **c** pereopod 3 (thread-spinning; see Additional file 2 for 3D movie). **d** pereopod 4 (non-thread-spinning; see Additional file 3 for 3D movie). Abbreviations: bm1 and bm2, basis muscles 1 and 2; cm1 and cm2, carpal muscles 1 and 2; mm1 and mm2, merus muscle 1 and 2; p1-p6, pereopod 1–6; pm1 and pm2, propodal muscles 1 and 2; tg1(p1), tg1(p2), tg1(p3), thoracic gland 1 at pereonites 1–3; tg2(p2), tg2(p3), thoracic gland 2 at pereonites 2 and 3. Scale bars, 0.5 mm for **a**, 30 μm **b**-**d**

The remarkable resolution of SBM-SEM images revealed many surprising features of the tanaid spinneret system (Fig. 1). Two secretory glands (thoracic gland 1 and 2) occur in the thoracic segments (pereonites) associated with the base of each of the three thread-spinning pereopods (Figs. 1b, 3a “tg1 and tg2”, Additional file 1). Each gland connects to a single duct, which delivers secretion all the way to the tip of the spinneret leg (Fig. 1b, Additional file 1). Each spinneret leg (pereopods 1–3) is associated with a specific pair of thoracic glands (tg1 and tg2), and a pair of ducts derived from these glands. Thoracic gland 1 (tg1) for each leg is highly elongated, extending posteriorly up to a length of 2 pereonites from the corresponding spinneret leg (Fig. 1b, Additional file 1). The ducts from the thoracic glands in the spinneret legs run alongside the leg nerves derived from central nervous system (Figs. 1b, c, 3b, Additional files 1, 2). These ducts travel through the middle of the leg between leg muscles, and parallel the leg nerves until they reach the distal end of the leg (Figs. 1c, 3c, e, Additional file 2). The ducts from the two thoracic glands (tg1 and tg2), although closely parallel, remain separate throughout the leg. Each segment on the spinneret legs has two muscles, interpreted as abductor and adductor muscles (Fig. 1c, Additional file 2). This configuration of morphological units is shared by all three spinneret legs (pereopods 1–3). The two glands we describe are possibly homologous with the glands reported by Siewing [15] in paratanaoid *Heterotanais oerstedii*: called "Intraperikardialer Drüsenteil" and "Extraperikardialer Drüsenteil", due to their locations and distinct shapes. Blanc [14] and Siewing [15] reported a duct derived from one of these glands that ran through a pereopod in *H. oerstedii*. This duct is presumably the same as one of the two ducts that we found in *Sinelobus* sp. These prior studies presumably overlooked a separation between the two ducts due to methodological limitations, because their interpretation that two glands exist but one lacks a duct seems improbable. Such a resolution problem might have been caused by the thickness of the histological sections.

Non-spinneret legs (pereopods 4–6) exhibit a similar morphology to spinneret legs except that they lack the secretory ducts: leg nerves pass among the muscles to innervate the distal end of the leg, and all leg segments have two muscles in a comparable position to those in spinneret legs (Figs. 1d, 3c, d, Additional file 3). Due to the shared spatial arrangement of these leg muscles between spinneret and non-spinneret legs, the leg muscles should be considered as homologous units among the legs. The remarkable enlargement of the propodal muscle 1 and merus muscle 1 (pm1 and mm1 in Fig. 1c) in the spinneret legs implies that they play an important role in the spinneret function of these legs. Only major nerves are reconstructed in the whole-leg scans (Fig. 1c, d) for clarity. The stripy pattern apparent in Fig. 3d is an artifact generated during the reconstruction process, which is a major disadvantage of SBF-SEM caused by electron charging of the non-conductive resin surrounding the specimen.

**Fig. 2:**
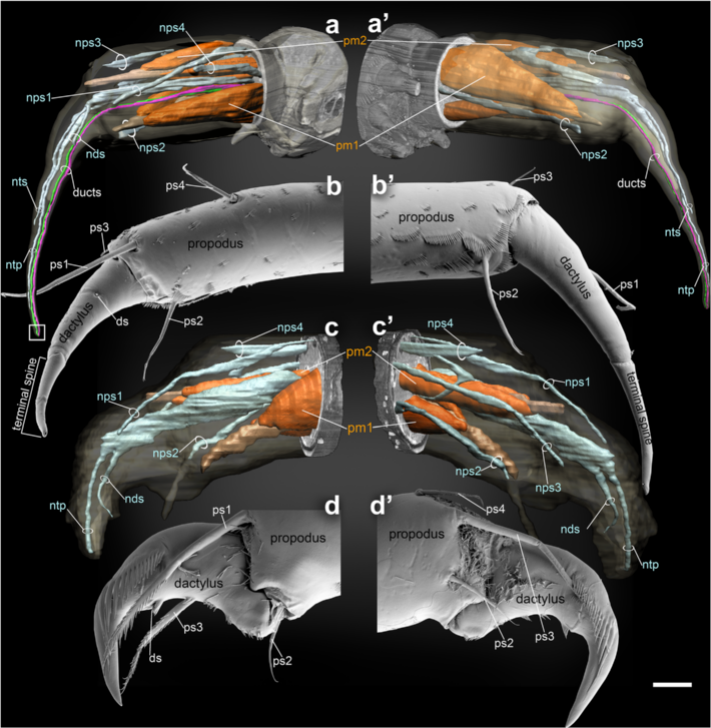
Detailed internal and external morphology of the leg tips of pereopods 3 and 4 of male *Sinelobus* sp. **a**, a' SBF-SEM scans showing the interior structure of the left pereopod 3 (thread-spinning), posterior and anterior view. **b**, b' conventional SEM images of left pereopod 3, posterior and anterior view. **c**, c' SBF-SEM scans showing the interior structure of left pereopod 4 (non-thread-spinning), posterior and anterior view. **d**, d' conventional SEM images of left pereopod 4, posterior and anterior view. Abbreviations: ds, dactylar setae; nds, nervous divisions innervating dactylar setae; nps1-4, nervous divisions innervating propodal setae 1–4; ntp, nervous divisions innervating terminal pore; nts, nervous divisions innervating base of terminal spine; pm1 and pm2, propodal muscles 1 and 2; ps1-ps4, propodal setae 1–4. The white square in **a** indicates the area shown in Fig. 4a, b. Scale bars, 15 μm for **a**, a’, c, c’, 10 μm for **b**, b’, d, d’

**Fig. 3:**
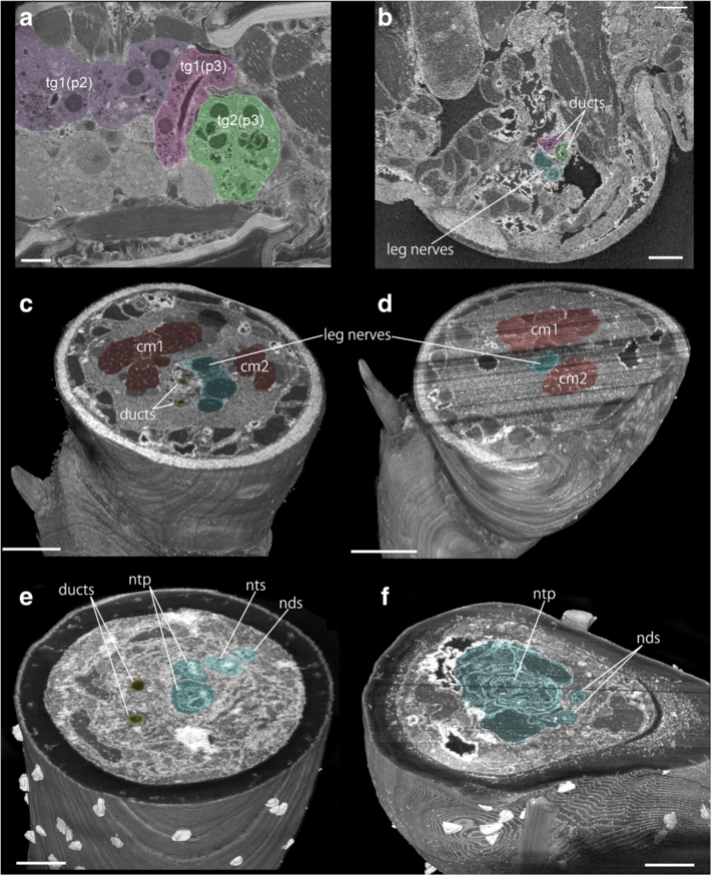
Reconstructed planes from an SBF-SEM scan showing detailed configuration of glands, nerves and ducts of male *Sinelobus* sp. **a** sagittal plane around pereonites 3–4 with thoracic glands highlighted. **b** sagittal plane at the base of the pereopod 3 (thread-spinning) with ducts and nerves highlighted. **c** transverse plane at the middle of the carpus of pereopod 3 with limb muscles and nerves highlighted. **d** transverse plane at the middle of the carpus of pereopod 4 (non-thread-spinning) with limb muscles and nerves highlighted. **e** transverse plane at the base of the dactylus of pereopod 3 with ducts and nerves highlighted. **f** transverse plane at the base of the dactylus of pereopod 4 with nerves highlighted. In panels **c**-**f** the remaining portion of the leg to be sectioned is visible below the most recently exposed section surface. Abbreviations as in Figs. 1, and 2. Scale bars, 10 μm for **a**-**d**, 2 μm for **e**, **f**

**Fig. 4:**
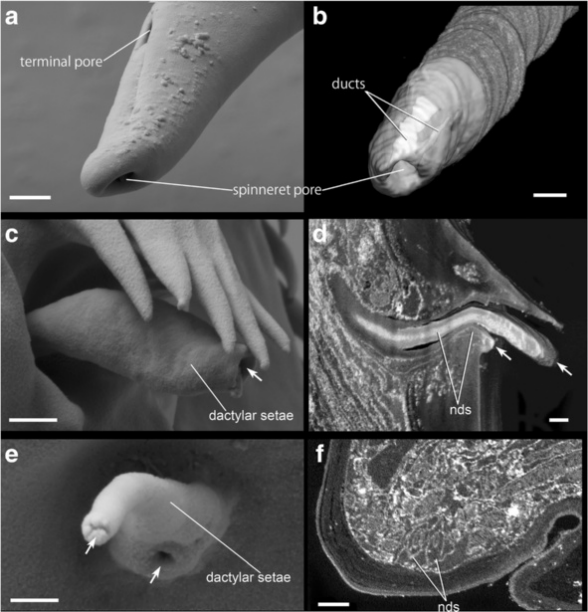
Detailed structure of the terminal spine and aesthetasc-like setae on the spinneret-leg tip of *Sinelobus* sp. **a** conventional SEM image of the distal end of the terminal spine of pereopod 3 (thread-spinning) (**b**) reconstructed terminal spine from SBF-SEM scans of pereopod 3 shows ducts merging at the tip. **c** conventional SEM image of the dactylar setae of pereopod 4 (non-thread-spinning). **d** reconstructed sagittal plane through the dactylar setae from SBF-SEM scans of pereopod 4. **e** conventional SEM image of the dactylar setae of pereopod 3. **f** transverse plane at the base of dactylar setae from a SBF-SEM scan of pereopod 3. Abbreviation: nds, nervous divisions innervating dactylar setae. Scale bars, 1 μm for **a**, **b**, 0.5 μm for **c**, **d**, 0.3 μm for **e**, **f**

Higher resolution SBM-SEM images of the leg tips revealed impressive details about the paths of the secretory ducts and the many nerves, and their relations to surface features on the leg like specific setae and pores (Figs. 2, 3e, f), which were not known from prior studies [18, 19]. These show striking differences between the spinneret legs (Fig. 2a, b) and the non-spinneret legs (Fig. 2c, d) related to their different functions. On the spinneret legs, the two separate ducts pass entirely through the propodus, dactylus and terminal spine and, along with the leg nerves, extend to the distal end of the spine (Figs. 2a, a’, 3e). These ducts fuse into one just before they reach the spinneret pore at the tip of terminal spine (Fig. 4a, b). This separation of ducts may indicate that two chemically different secretory products are mixed at the tip, presumably to produce optimized material properties of the thread.

Spinneret and non-spinneret legs also exhibited some intriguing differences in setation. On both spinneret and non-spinneret legs, four setae occur on the propodus, and one aesthetasc-like setae on the dactylus (ps1-4 and ds in Fig. 2). Setae were located as follows: a) propodal setae 1 (ps1)- dorsoposterior side of the mid part of the propodus, b) propodal setae 2 (ps2)- anteroventral side of the distal part of the propodus, c) propodal setae 3 (ps3)- dorsoanterior side of the distal part of propodus, d) propodal setae 4 (ps4)- dorsal side of the middle of propodus, e) dactylar setae (ds)- ventral side of the dactylus. These nearly identical setal locations on pereopods 3 and 4 suggest that they are all serially homologous. However, remarkable morphological differences in form exist between propodal setae 3 and the dactylar setae between pereopods 3 and 4 (Fig. 2b, b’, d, d’). These propodal setae 1–4 could be homologous with the seta described in *Sinelobus barretti* by Edgar [18], however the dactylar setae we report here were never noted before, as far as we know. However, it is not surprising that such tiny setae may have been overlooked before, since most tanaid studies were conducted for taxonomic purpose using light microscopy, not for high-resolution morphology.

**Fig. 5:**
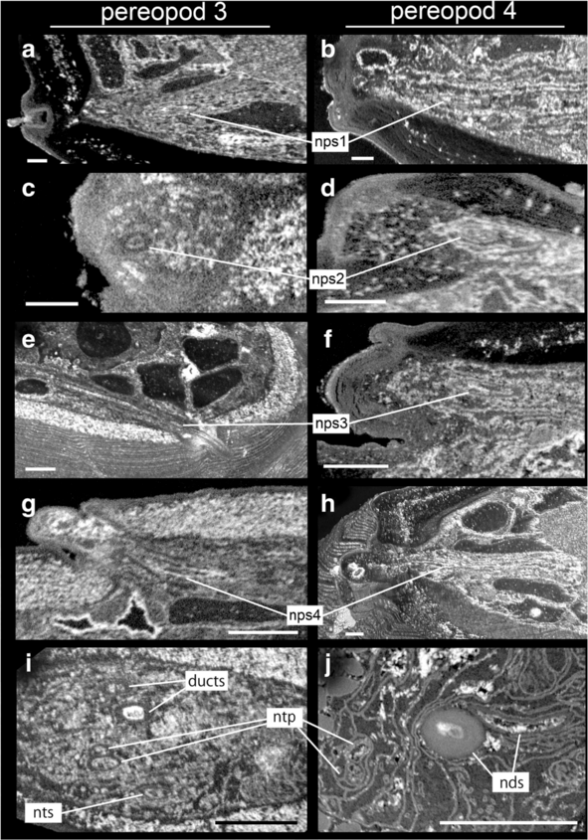
Nanometer resolution images of outer dendritic cilia in the dactyl terminal-spine area from SBF-SEM scans of spinneret (pereopod 3) and non-spinneret (pereopod 4) legs of *Sinelobus* sp. **a**, **b** coronal plane at the base of propodal seta 1. **c**, **d** coronal plane at the base of propodal seta 2. **e**, **f** coronal plane at the base of propodal seta 3. **g**, **h** coronal plane at the base of propodal seta 4. **i**, **j** transverse plane at the base of the dactylus. Abbreviations as in Fig. 2. Scale bars, 300 nm

SBM-SEM images of the leg tips also revealed in great detail the relation of nerves to individual setae (Fig. 2a, a’, c, c’). Each seta noted above is innervated by an individual nerve branch. Most setae have only single-cilium innervation (nps1-4 and nts in Fig. 5), but exceptionally, the nerve branch innervating the terminal pore (at the tip of the terminal spine) and dactylar setae innervates two separated cilia (ntp in Fig. 5i, j and nds in Fig. 4d, f). The shared innervation pattern of the nds cilia in pereopods 3 and 4 (in both legs, cilia are innervated at the base and the distal end in dactylar setae; Fig. 4d, e), suggests that they have similar function, probably bimodal, like a chemo-mechanosensory aesthetasc [20, 21].

Because of the common arrangement of setae, the nerves in the distal part of both pereopods 3 and 4 are quite similar (Fig. 2a, a’, c, c’). However a small branch of the nerve innervating base of terminal spine (nts in Fig. 2a) occurs only in pereopods 3, which implies that the spinneret leg has specific adaptations to ensure precise motor control of the terminal spine. These setal and nerve arrangements in pereopods 3 and 4 are representative of the series of spinneret and non-spinneret legs respectively.

The spinneret system described here seems unlikely to be homologous with any other crustacean spinneret systems, based on current knowledge. *Phoxokalliapseudes tomiokaensis*, the most basal linage of silk-spinning Tanaidacea, possesses several types of spinneret systems throughout pereiopods 1–6 [13]. However, these glands are located in the legs, not in the pereonites, so they are not likely homologous with any glands in *Sinelobus* sp. Some other peracarids (e.g., Amphipoda), possess spinneret systems on pereiopods 3 and 4 [22, 23]. But several separated glands are located on the legs, which suggests that they are not homologous with the single-gland arrangement of *Sinelobus* sp. However, as seen in *Sinelobus* sp., the amphipod spinneret ducts merge into one before they reach the spinneret pore opening, so the physical and chemical properties presumably change in a similar way before release into the water [22]. Podocopid ostracods also possess a gland in the forehead at the base of their antenna and a duct running though the antennal exopod toward the tip of the exopod [24], but this arrangement also differs significantly from the spinneret system in *Sinelobus* sp. Unfortunately, critical information is lacking about the presence and ontogeny of spinneret systems in other tanaids, and in other crustacean groups, to conclude with confidence how often spinneret systems have evolved independently in the Crustacea.

## Conclusions

This first comprehensive description of the spinneret system in a tanaid provides new evidence for a distinctive morphology: two separate ducts, derived from two separated glands in each thread-spinning segment, that merge at the leg tip or the terminal spine before secretions are released. This arrangement seems to be unique to the Tanaidomorpha, as there are no evident homologous systems in other Crustacea. However, far too little is known about other taxa — including development, detailed histology, and detailed three dimensional fine-structure — to draw any broad generalizations. To better understand the evolution of spinneret systems in Crustacea, a more extensive survey of new and previously studied species using more powerful visualization tools like SBF-SEM would be required.

Our results demonstrate the dramatic advantage of SBF-SEM for descriptions of mesoscale morphology (in our case, a few body-segments wide) at a nanoscopic resolution (e.g., single dendrite-scale), particularly for small-bodied animals. Tremendous morphological disparity exists among microscopic animals (e.g., meiofauna, [25]), whose nanoscopic 3D anatomy has largely been obscured using conventional microscopy. SBF-SEM offers a powerful new tool for studying these challenging animals as part of the renaissance of morphology.

## Additional files

Additional file 1: Overall morphology of glands, ducts and nerves of the left-half of pereonites 3–4 of *Sinelobus* sp. This movie corresponds to Fig. 1b. (M4V 19194 kb) Additional file 2: Ducts, muscles and nerves in the pereopod 3 (spinneret legs) of *Sinelobus* sp. This movie corresponds to Fig. 1c. (M4V 7782 kb) Additional file 3: Ducts, muscles and nerves in the pereopod 4 (non-spinneret legs) of *Sinelobus* sp. This movie corresponds to Fig. 1d. (M4V 7103 kb)

## Abbreviations

bm1 and 2: basis muscles 1 and 2
cm1 and 2: carpal muscles 1 and 2
ds: dactylar setae
mm1 and 2: merus muscles 1 and 2
nds: nervous divisions innervating dactylar setae
nps1-4: nervous divisions innervating propodal setae 1–4
ntp: nervous divisions innervating terminal pore
nts: nervous divisions innervating base of terminal spine
p1-6: pereopods 1–6
pm1 and 2: propodal muscles 1 and 2
ps1-4: propodal setae 1–4.
SBF: serial block face
SEM: scanning electron microscopy
tg1(p1-3): thoracic gland 1 at pereonites 1–3
tg2(p2, 3): thoracic gland 2 at pereonites 2 and 3
